# Supplementary material for: Simulations predict stronger CRISPRi transcriptional repression in plants for identical than heterogeneous gRNA target sites
Source: Synth Biol (Oxf). 2025 Apr 18;10(1):ysae020. doi: 10.1093/synbio/ysae020 (PMC12007490; doi:10.1093/synbio/ysae020)
Supplement: ysae020_Supp [file ysae020_supp.zip › suppl_data/Supplementary_Material.pdf]

Simulations Predict Stronger CRISPRi Transcriptional Repression in Plants for Identical than Heterogeneous gRNA Target Site
Supplementary S1: Derivation of Equations and Parameter Perturbation for CRISPRi Gene Expression Repression

Helen Scott[1], Alessandro Occhialini[2,3], Scott C. Lenaghan[3,4], and Jacob Beal[1]

[1]Intelligent Software and Systems, RTX BBN Technologies, Cambridge, MA 02138, USA
[2]Department of Plant Sciences, University of Tennessee, 112 Plant Biotechnology Building 2505 E J Chapman Drive, Knoxville, TN 37996, USA
[3]Center for Agricultural Synthetic Biology (CASB), University of Tennessee, 2640 Morgan Circle Dr., Knoxville, TN 37996, USA
[4]Department of Food Science, University of Tennessee Institute of Agriculture (UTIA), 102 Food Safety and Processing Building 2600 River Dr., Knoxville TN 37996, USA

October 29, 2024

Contents

1 Organization **2**

2 Model Construction **2**
- 2.1 Core Biological Mechanisms: Transcription, Translation, Degradation, and Dilution 2
- 2.2 dCas9 Complex Kinetics 3
- 2.3 Control Mechanisms: Transcription Factor Regulation 4

3 Circuit Models **5**
- 3.1 0 Target Sites 6
- 3.2 1 Target Site 6
- 3.3 2 Heterogeneous Target Sites 6
- 3.4 2 Identical Target Sites 6
- 3.5 3 Heterogeneous Target Sites 7
- 3.6 3 Identical Target Sites 7
- 3.7 4 Heterogeneous Target Sites 8
- 3.8 4 Identical Target Sites 8

3.9 5 Heterogeneous Target Sites 8
3.10 5 Identical Target Sites 9
3.11 6 Heterogeneous Target Sites 9
3.12 6 Identical Target Sites 10

4 Parameter Fitting **11**

5 Analysis of Repression Sensitivity **13**
5.1 Sensitivity to Parameter Values 13
5.1.1 Multi-Parameter Perturbations 15

1 Organization

This document presents the equations used for modeling the various CRISPRi promoter architectures. Section 2 discusses the derivation of the differential equation models used for each individual component in the circuits. Section 3 then composes these elements into all of the promoter architectures investigated. Section 4 identifies the parameter values used in the models and the sources for those parameters. Section 5 then analyzes the sensitivity of the model to those parameter values.

Table S1 presents the variables used throughout this document, using the following typical conventions:

- Molecule counts are upper case, with the class of molecule indicated by latter (e.g., G for gRNA, P for protein)
- Particular species are indicated by subscript (e.g., G_i indicates a specific gRNA).
- Rate constants are lower case Greek, except k for reaction rate constants.
- Hill-Langmuir equations use n for coefficient and K_R for dissociation constants.

2 Model Construction

The possible repression circuits considered here are made of several core biological mechanisms and repeating control mechanisms. We model each mechanism with a standard differential equation model, selected for modularity and abstraction in order to keep the number of required parameters as low as possible. The mechanisms considered are: genetic production by transcription and translation from the engineered vector and the degradation or dilution of products over time, CRISPR complex formation and, and transcription factor regulation of expression.

2.1 Core Biological Mechanisms: Transcription, Translation, Degradation, and Dilution

We consider two classes of products, gRNA and proteins. For a gRNA species G_i, its production depends only on the rate of transcription from copies of the vector. Decay is assumed to be the standard exponential and dilution negligible (i.e., non-dividing cells). This can be expressed as:

$$\frac{\mathrm{d}[G_i]}{\mathrm{d}t} = \alpha_{r,i} V - \delta_g [G_i] \tag{1}$$

Where G_i is the the concentration of the RNA product, $\alpha_{r,i}$ the transcription rate of the controlling promoter in the absence of regulation, and δ_g the degradation rate for unbound gRNA. All symbols are also defined in Table S1.

Variable	Meaning
V_i	Construct vector, species i
G_i	gRNA, species i
P_{dCas9}	dCas9 protein species
P_{GFP}	GFP protein species
$C_{\text{dCas9},i}$	Complex of dCas9 protein and gRNAi
$\alpha_{r,i}$	Transcription rate, species i
$\alpha_{p,i}$	Coupled transcription and translation rate, species i
$\alpha_{p,i}^0$	Maximal coupled transcription and translation rate, species i
δ_g	gRNA degradation rate
λ	Stable Molecule Dilution Rate
k_{C_g}	dCas9 and gRNA binding rate
n	Hill coefficient
K_A	Hill Activation Constant
K_R	Hill Repression Constant
m	Number of Identical Binding Sites

Table S1: Table of variable symbols and meanings

For protein products, the rate of formation of the product is dependent on both transcription and translation. Transcription and translation are modeled as a joint rate in the model under the assumption that mRNA decay rate is fast compared to decay rate of the proteins under consideration, which allows the intermediate stage of mRNA to be abstracted away, reducing the number of parameters. In the longer time series for the constitutive expression of GFP (Figure S1), the constant rate of decrease of GFP from hours 64 to 200 is indicative of stable molecule dilution due to cell division, rather than protein degradation. With the assumption that protein molecules are otherwise stable over the time course, Production and dilution of a protein can thus be expressed as:

$$\frac{\mathrm{d}[P_i]}{\mathrm{d}t} = \alpha_{p,i}V - \lambda[P_i] \tag{2}$$

Where $[P_i]$ is the the concentration of the RNA product, $\alpha_{p,i}$ the joint production rate from transcription and translation in the absence of regulation, and λ is the stable molecule dilution rate for the protein.

2.2 dCas9 Complex Kinetics

The differential equation system for all models incudes a reaction for the binding of Cas9 and gRNA into an active complex. While there are many other factors that can play into CRISPR kinetics, as these are not anticipated as being limited factors or being manipulated with regards to the specific system under investigation. We retain the binding, however, as the degradation rate of gRNA can be large with respect to the binding rate when expression levels of Cas9 and gRNA are low, which is especially relevant in the cases of many heterogeneous sites, as the concentration of any given gRNA will be low. When the gRNA

binds to Cas9 protein, the resulting complex is significantly more stable, and is not expected to experience a significant rate of dissociation. Complex formation may thus be described as the reaction:

$$P_{\text{dCas9}} + G_i \xrightarrow{k_{C_g}} C_{\text{dCas9},i} \tag{3}$$

Where P_{dCas9} is the dCas9 protein, G_i is a particular gRNA, $C_{\text{dCas9},i}$ is the complex formed by the binding of these two molecules and k_{C_g} is the reaction rate constant. This complex is affected by the same stable molecule diffusion as proteins, thus the following dynamics describe dCas9 complex binding.

$$\frac{\mathrm{d}[P_{\text{dCas9}}]}{\mathrm{d}t} = \alpha_{p,\text{dCas9}} V - \lambda[P_{\text{dCas9}}] - \sum_i k_{C_g}[P_{\text{dCas9}}][G_i] \tag{4}$$

$$\frac{\mathrm{d}[G_i]}{\mathrm{d}t} = \alpha_{r,i} V - \delta_g[G_i] - k_{C_g}[P_{\text{dCas9}}][G_i] \tag{5}$$

$$\frac{\mathrm{d}[C_{\text{dCas9},i}]}{\mathrm{d}t} = k_{C_g}[P_{\text{dCas9}}][G_i] - \lambda[C_{\text{dCas9},i}] \tag{6}$$

2.3 Control Mechanisms: Transcription Factor Regulation

The activity of gRNA-guided dCas9 transcription factors is analogous to other types of transcription factor regulation, and so we modeled the effect of regulation on gene production using the standard Hill-Langmuir equation [1]. Specifically, given the models of production given above, we model transcriptional regulation as a modulation of the effective transcription rate $\alpha_{r,i}$ or joint transcription/translation rate, $\alpha_{p,i}$.

As the production of a given species, i is repressed by the dCas9 complex, $C_{\text{dCas9},i}$, then we model the modulation of the production rate in terms of the concentration of the transcription factor as:

$$\alpha_{p,i} = \alpha^0_{p,i} \frac{(K_R)^n}{(K_R)^n + [C_{\text{dCas9},i}]^n} \tag{7}$$

Where $\alpha^0_{p,i}$ is the maximal production rate, n is the Hill coefficient, and K_R is the Hill Repression constant.

When there are multiple binding sites, their effect on the target is multiplicative, so that each unique binding site has its own Hill-Langmuir term [1].

$$\alpha_{p,i} = \alpha^0_{p,i} \prod_i \frac{(K_R)^n}{(K_R)^n + [C_{\text{dCas9},i}]^n} \tag{8}$$

In the case of identical binding sites, where the same dCas9-gRNA complex concentration is used for all of the Hill-Langmuir terms, this can be simplified to:

$$\alpha_{p,i} = \alpha^0_{p,i} \left(\frac{(K_R)^n}{(K_R)^n + [C_{\text{dCas9},i}]^n} \right)^m \tag{9}$$

where m is the total number of identical binding sites.

In both of these cases, we assume no interference between the gRNA target sites (e.g. like that which could be caused do to overlapping binding sites). To incorporate this interference, the concentration of the dCas9-gRNA complex could be modulated to reflect the effective concentration.

3 Circuit Models

From these components, models for all possible repressible promoters can be generated by connecting the equations above together and repeating the Hill-Langmuir terms the desired number of times. Models for systems with up to 6 identical or heterogeneous target sites are included below.

3.1 0 Target Sites

$$\frac{\mathrm{d}V_2}{\mathrm{d}t} = -\lambda V_2 \tag{10}$$
$$\frac{\mathrm{d}[P_{\mathrm{dCas9}}]}{\mathrm{d}t} = \alpha_{p,P_{\mathrm{dCas9}}} V_2 - \lambda [P_{\mathrm{dCas9}}] \tag{11}$$
$$\frac{\mathrm{d}[P_{\mathrm{GFP}}]}{\mathrm{d}t} = \alpha_{p,P_{\mathrm{GFP}}} V_2 - \lambda [P_{\mathrm{GFP}}] \tag{12}$$

3.2 1 Target Site

$$\frac{\mathrm{d}V_2}{\mathrm{d}t} = -\lambda V_2 \tag{13}$$
$$\frac{\mathrm{d}[G_1]}{\mathrm{d}t} = \alpha_{r,G_1} V_1 - k_{C_g}[G_1][P_{\mathrm{dCas9}}] - \delta_g [G_1] \tag{14}$$
$$\frac{\mathrm{d}[C_{\mathrm{dCas9},1}]}{\mathrm{d}t} = k_{C_g}[G_1][P_{\mathrm{dCas9}}] - \lambda [C_{\mathrm{dCas9},1}] \tag{15}$$
$$\frac{\mathrm{d}[P_{\mathrm{dCas9}}]}{\mathrm{d}t} = \alpha_{p,P_{\mathrm{dCas9}}} V_2 - \lambda [P_{\mathrm{dCas9}}] - k_{C_g}[G_1][P_{\mathrm{dCas9}}] \tag{16}$$
$$\frac{\mathrm{d}[P_{\mathrm{GFP}}]}{\mathrm{d}t} = \alpha^0_{p,P_{\mathrm{GFP}}} \frac{(K_R)^n}{(K_R)^n + [C_{\mathrm{dCas9},1}]^n} V_2 - \lambda [P_{\mathrm{GFP}}] \tag{17}$$
$$\frac{\mathrm{d}V_1}{\mathrm{d}t} = -\lambda V_1 \tag{18}$$

3.3 2 Heterogeneous Target Sites

$$\frac{\mathrm{d}V_2}{\mathrm{d}t} = -\lambda V_2 \tag{19}$$
$$\frac{\mathrm{d}[G_1]}{\mathrm{d}t} = \alpha_{r,G_1} V_1 - k_{C_g}[G_1][P_{\mathrm{dCas9}}] - \delta_g [G_1] \tag{20}$$
$$\frac{\mathrm{d}[C_{\mathrm{dCas9},1}]}{\mathrm{d}t} = k_{C_g}[G_1][P_{\mathrm{dCas9}}] - \lambda [C_{\mathrm{dCas9},1}] \tag{21}$$
$$\frac{\mathrm{d}[G_2]}{\mathrm{d}t} = \alpha_{r,G_2} V_1 - k_{C_g}[G_2][P_{\mathrm{dCas9}}] - \delta_g [G_2] \tag{22}$$
$$\frac{\mathrm{d}[C_{\mathrm{dCas9},2}]}{\mathrm{d}t} = k_{C_g}[G_2][P_{\mathrm{dCas9}}] - \lambda [C_{\mathrm{dCas9},2}] \tag{23}$$
$$\frac{\mathrm{d}[P_{\mathrm{dCas9}}]}{\mathrm{d}t} = \alpha_{p,P_{\mathrm{dCas9}}} V_2 - \lambda [P_{\mathrm{dCas9}}] - k_{C_g}[G_1][P_{\mathrm{dCas9}}] - k_{C_g}[G_2][P_{\mathrm{dCas9}}] \tag{24}$$
$$\frac{\mathrm{d}[P_{\mathrm{GFP}}]}{\mathrm{d}t} = \alpha^0_{p,P_{\mathrm{GFP}}} \frac{(K_R)^n}{(K_R)^n + [C_{\mathrm{dCas9},1}]^n} \frac{(K_R)^n}{(K_R)^n + [C_{\mathrm{dCas9},2}]^n} V_2 - \lambda [P_{\mathrm{GFP}}] \tag{25}$$
$$\frac{\mathrm{d}V_1}{\mathrm{d}t} = -\lambda V_1 \tag{26}$$

3.4 2 Identical Target Sites

$$\frac{\mathrm{d}V_2}{\mathrm{d}t} = -\lambda V_2 \tag{27}$$
$$\frac{\mathrm{d}[G_1]}{\mathrm{d}t} = \alpha_{r,G_1} V_1 - k_{C_g}[G_1][P_{\mathrm{dCas9}}] - \delta_g [G_1] \tag{28}$$

$$\frac{\mathrm{d}[C_{\mathrm{dCas9},1}]}{\mathrm{d}t} = k_{C_g}[G_1][P_{\mathrm{dCas9}}] - \lambda[C_{\mathrm{dCas9},1}] \tag{29}$$

$$\frac{\mathrm{d}[P_{\mathrm{dCas9}}]}{\mathrm{d}t} = \alpha_{p,P_{\mathrm{dCas9}}}V_2 - \lambda[P_{\mathrm{dCas9}}] - k_{C_g}[G_1][P_{\mathrm{dCas9}}] \tag{30}$$

$$\frac{\mathrm{d}[P_{\mathrm{GFP}}]}{\mathrm{d}t} = \alpha^0_{p,P_{\mathrm{GFP}}}\frac{(K_R)^n}{(K_R)^n + [C_{\mathrm{dCas9},1}]^n}\frac{(K_R)^n}{(K_R)^n + [C_{\mathrm{dCas9},1}]^n}V_2 - \lambda[P_{\mathrm{GFP}}] \tag{31}$$

$$\frac{\mathrm{d}V_1}{\mathrm{d}t} = -\lambda V_1 \tag{32}$$

3.5 3 Heterogeneous Target Sites

$$\frac{\mathrm{d}V_2}{\mathrm{d}t} = -\lambda V_2 \tag{33}$$

$$\frac{\mathrm{d}[G_1]}{\mathrm{d}t} = \alpha_{r,G_1}V_1 - k_{C_g}[G_1][P_{\mathrm{dCas9}}] - \delta_g[G_1] \tag{34}$$

$$\frac{\mathrm{d}[C_{\mathrm{dCas9},1}]}{\mathrm{d}t} = k_{C_g}[G_1][P_{\mathrm{dCas9}}] - \lambda[C_{\mathrm{dCas9},1}] \tag{35}$$

$$\frac{\mathrm{d}[G_2]}{\mathrm{d}t} = \alpha_{r,G_2}V_1 - k_{C_g}[G_2][P_{\mathrm{dCas9}}] - \delta_g[G_2] \tag{36}$$

$$\frac{\mathrm{d}[C_{\mathrm{dCas9},2}]}{\mathrm{d}t} = k_{C_g}[G_2][P_{\mathrm{dCas9}}] - \lambda[C_{\mathrm{dCas9},2}] \tag{37}$$

$$\frac{\mathrm{d}[G_3]}{\mathrm{d}t} = \alpha_{r,G_3}V_1 - k_{C_g}[G_3][P_{\mathrm{dCas9}}] - \delta_g[G_3] \tag{38}$$

$$\frac{\mathrm{d}[C_{\mathrm{dCas9},3}]}{\mathrm{d}t} = k_{C_g}[G_3][P_{\mathrm{dCas9}}] - \lambda[C_{\mathrm{dCas9},3}] \tag{39}$$

$$\frac{\mathrm{d}[P_{\mathrm{dCas9}}]}{\mathrm{d}t} = \alpha_{p,P_{\mathrm{dCas9}}}V_2 - \lambda[P_{\mathrm{dCas9}}] - k_{C_g}[G_1][P_{\mathrm{dCas9}}] - k_{C_g}[G_2][P_{\mathrm{dCas9}}] - k_{C_g}[G_3][P_{\mathrm{dCas9}}] \tag{40}$$

$$\frac{\mathrm{d}[P_{\mathrm{GFP}}]}{\mathrm{d}t} = \alpha^0_{p,P_{\mathrm{GFP}}}\frac{(K_R)^n}{(K_R)^n + [C_{\mathrm{dCas9},1}]^n}\frac{(K_R)^n}{(K_R)^n + [C_{\mathrm{dCas9},2}]^n}\frac{(K_R)^n}{(K_R)^n + [C_{\mathrm{dCas9},3}]^n}V_2 - \lambda[P_{\mathrm{GFP}}] \tag{41}$$

$$\frac{\mathrm{d}V_1}{\mathrm{d}t} = -\lambda V_1 \tag{42}$$

3.6 3 Identical Target Sites

$$\frac{\mathrm{d}V_2}{\mathrm{d}t} = -\lambda V_2 \tag{43}$$

$$\frac{\mathrm{d}[G_1]}{\mathrm{d}t} = \alpha_{r,G_1}V_1 - k_{C_g}[G_1][P_{\mathrm{dCas9}}] - \delta_g[G_1] \tag{44}$$

$$\frac{\mathrm{d}[C_{\mathrm{dCas9},1}]}{\mathrm{d}t} = k_{C_g}[G_1][P_{\mathrm{dCas9}}] - \lambda[C_{\mathrm{dCas9},1}] \tag{45}$$

$$\frac{\mathrm{d}[P_{\mathrm{dCas9}}]}{\mathrm{d}t} = \alpha_{p,P_{\mathrm{dCas9}}}V_2 - \lambda[P_{\mathrm{dCas9}}] - k_{C_g}[G_1][P_{\mathrm{dCas9}}] \tag{46}$$

$$\frac{\mathrm{d}[P_{\mathrm{GFP}}]}{\mathrm{d}t} = \alpha^0_{p,P_{\mathrm{GFP}}}\frac{(K_R)^n}{(K_R)^n + [C_{\mathrm{dCas9},1}]^n}\frac{(K_R)^n}{(K_R)^n + [C_{\mathrm{dCas9},1}]^n}\frac{(K_R)^n}{(K_R)^n + [C_{\mathrm{dCas9},1}]^n}V_2 - \lambda[P_{\mathrm{GFP}}] \tag{47}$$

$$\frac{\mathrm{d}V_1}{\mathrm{d}t} = -\lambda V_1 \tag{48}$$

3.7 4 Heterogeneous Target Sites

$$\frac{\mathrm{d}V_2}{\mathrm{d}t} = -\lambda V_2 \tag{49}$$

$$\frac{\mathrm{d}[G_1]}{\mathrm{d}t} = \alpha_{r,G_1} V_1 - k_{C_g}[G_1][P_{\mathrm{dCas9}}] - \delta_g [G_1] \tag{50}$$

$$\frac{\mathrm{d}[C_{\mathrm{dCas9},1}]}{\mathrm{d}t} = k_{C_g}[G_1][P_{\mathrm{dCas9}}] - \lambda [C_{\mathrm{dCas9},1}] \tag{51}$$

$$\frac{\mathrm{d}[G_2]}{\mathrm{d}t} = \alpha_{r,G_2} V_1 - k_{C_g}[G_2][P_{\mathrm{dCas9}}] - \delta_g [G_2] \tag{52}$$

$$\frac{\mathrm{d}[C_{\mathrm{dCas9},2}]}{\mathrm{d}t} = k_{C_g}[G_2][P_{\mathrm{dCas9}}] - \lambda [C_{\mathrm{dCas9},2}] \tag{53}$$

$$\frac{\mathrm{d}[G_3]}{\mathrm{d}t} = \alpha_{r,G_3} V_1 - k_{C_g}[G_3][P_{\mathrm{dCas9}}] - \delta_g [G_3] \tag{54}$$

$$\frac{\mathrm{d}[C_{\mathrm{dCas9},3}]}{\mathrm{d}t} = k_{C_g}[G_3][P_{\mathrm{dCas9}}] - \lambda [C_{\mathrm{dCas9},3}] \tag{55}$$

$$\frac{\mathrm{d}[G_4]}{\mathrm{d}t} = \alpha_{r,G_4} V_1 - k_{C_g}[G_4][P_{\mathrm{dCas9}}] - \delta_g [G_4] \tag{56}$$

$$\frac{\mathrm{d}[C_{\mathrm{dCas9},4}]}{\mathrm{d}t} = k_{C_g}[G_4][P_{\mathrm{dCas9}}] - \lambda [C_{\mathrm{dCas9},4}] \tag{57}$$

$$\frac{\mathrm{d}[P_{\mathrm{dCas9}}]}{\mathrm{d}t} = \alpha_{p,P_{\mathrm{dCas9}}} V_2 - \lambda [P_{\mathrm{dCas9}}] - k_{C_g}[G_1][P_{\mathrm{dCas9}}] - k_{C_g}[G_2][P_{\mathrm{dCas9}}] - k_{C_g}[G_3][P_{\mathrm{dCas9}}] - k_{C_g}[G_4][P_{\mathrm{dCas9}}] \tag{58}$$

$$\frac{\mathrm{d}[P_{\mathrm{GFP}}]}{\mathrm{d}t} = \alpha^0_{p,P_{\mathrm{GFP}}} \frac{(K_R)^n}{(K_R)^n + [C_{\mathrm{dCas9},1}]^n} \frac{(K_R)^n}{(K_R)^n + [C_{\mathrm{dCas9},2}]^n} \frac{(K_R)^n}{(K_R)^n + [C_{\mathrm{dCas9},3}]^n} \frac{(K_R)^n}{(K_R)^n + [C_{\mathrm{dCas9},4}]^n} V_2 - \lambda [P_{\mathrm{GFP}}] \tag{59}$$

$$\frac{\mathrm{d}V_1}{\mathrm{d}t} = -\lambda V_1 \tag{60}$$

3.8 4 Identical Target Sites

$$\frac{\mathrm{d}V_2}{\mathrm{d}t} = -\lambda V_2 \tag{61}$$

$$\frac{\mathrm{d}[G_1]}{\mathrm{d}t} = \alpha_{r,G_1} V_1 - k_{C_g}[G_1][P_{\mathrm{dCas9}}] - \delta_g [G_1] \tag{62}$$

$$\frac{\mathrm{d}[C_{\mathrm{dCas9},1}]}{\mathrm{d}t} = k_{C_g}[G_1][P_{\mathrm{dCas9}}] - \lambda [C_{\mathrm{dCas9},1}] \tag{63}$$

$$\frac{\mathrm{d}[P_{\mathrm{dCas9}}]}{\mathrm{d}t} = \alpha_{p,P_{\mathrm{dCas9}}} V_2 - \lambda [P_{\mathrm{dCas9}}] - k_{C_g}[G_1][P_{\mathrm{dCas9}}] \tag{64}$$

$$\frac{\mathrm{d}[P_{\mathrm{GFP}}]}{\mathrm{d}t} = \alpha^0_{p,P_{\mathrm{GFP}}} \frac{(K_R)^n}{(K_R)^n + [C_{\mathrm{dCas9},1}]^n} \frac{(K_R)^n}{(K_R)^n + [C_{\mathrm{dCas9},1}]^n} \frac{(K_R)^n}{(K_R)^n + [C_{\mathrm{dCas9},1}]^n} \frac{(K_R)^n}{(K_R)^n + [C_{\mathrm{dCas9},1}]^n} V_2 - \lambda [P_{\mathrm{GFP}}] \tag{65}$$

$$\frac{\mathrm{d}V_1}{\mathrm{d}t} = -\lambda V_1 \tag{66}$$

3.9 5 Heterogeneous Target Sites

$$\frac{\mathrm{d}V_2}{\mathrm{d}t} = -\lambda V_2 \tag{67}$$

$$\frac{\mathrm{d}[G_1]}{\mathrm{d}t} = \alpha_{r,G_1} V_1 - k_{C_g}[G_1][P_{\mathrm{dCas9}}] - \delta_g [G_1] \tag{68}$$

$$\frac{\mathrm{d}[C_{\mathrm{dCas9},1}]}{\mathrm{d}t} = k_{C_g}[G_1][P_{\mathrm{dCas9}}] - \lambda[C_{\mathrm{dCas9},1}] \tag{69}$$

$$\frac{\mathrm{d}[G_2]}{\mathrm{d}t} = \alpha_{r,G_2}V_1 - k_{C_g}[G_2][P_{\mathrm{dCas9}}] - \delta_g[G_2] \tag{70}$$

$$\frac{\mathrm{d}[C_{\mathrm{dCas9},2}]}{\mathrm{d}t} = k_{C_g}[G_2][P_{\mathrm{dCas9}}] - \lambda[C_{\mathrm{dCas9},2}] \tag{71}$$

$$\frac{\mathrm{d}[G_3]}{\mathrm{d}t} = \alpha_{r,G_3}V_1 - k_{C_g}[G_3][P_{\mathrm{dCas9}}] - \delta_g[G_3] \tag{72}$$

$$\frac{\mathrm{d}[C_{\mathrm{dCas9},3}]}{\mathrm{d}t} = k_{C_g}[G_3][P_{\mathrm{dCas9}}] - \lambda[C_{\mathrm{dCas9},3}] \tag{73}$$

$$\frac{\mathrm{d}[G_4]}{\mathrm{d}t} = \alpha_{r,G_4}V_1 - k_{C_g}[G_4][P_{\mathrm{dCas9}}] - \delta_g[G_4] \tag{74}$$

$$\frac{\mathrm{d}[C_{\mathrm{dCas9},4}]}{\mathrm{d}t} = k_{C_g}[G_4][P_{\mathrm{dCas9}}] - \lambda[C_{\mathrm{dCas9},4}] \tag{75}$$

$$\frac{\mathrm{d}[G_5]}{\mathrm{d}t} = \alpha_{r,G_5}V_1 - k_{C_g}[G_5][P_{\mathrm{dCas9}}] - \delta_g[G_5] \tag{76}$$

$$\frac{\mathrm{d}[C_{\mathrm{dCas9},5}]}{\mathrm{d}t} = k_{C_g}[G_5][P_{\mathrm{dCas9}}] - \lambda[C_{\mathrm{dCas9},5}] \tag{77}$$

$$\frac{\mathrm{d}[P_{\mathrm{dCas9}}]}{\mathrm{d}t} = \alpha_{p,P_{\mathrm{dCas9}}}V_2 - \lambda[P_{\mathrm{dCas9}}] - k_{C_g}[G_1][P_{\mathrm{dCas9}}] - k_{C_g}[G_2][P_{\mathrm{dCas9}}] - k_{C_g}[G_3][P_{\mathrm{dCas9}}] - k_{C_g}[G_4][P_{\mathrm{dCas9}}] - k_{C_g}[G_5][P_{\mathrm{dCas9}}] \tag{78}$$

$$\frac{\mathrm{d}[P_{\mathrm{GFP}}]}{\mathrm{d}t} = \alpha^0_{p,P_{\mathrm{GFP}}}\frac{(K_R)^n}{(K_R)^n+[C_{\mathrm{dCas9},1}]^n}\frac{(K_R)^n}{(K_R)^n+[C_{\mathrm{dCas9},2}]^n}\frac{(K_R)^n}{(K_R)^n+[C_{\mathrm{dCas9},3}]^n}\frac{(K_R)^n}{(K_R)^n+[C_{\mathrm{dCas9},4}]^n}\frac{(K_R)^n}{(K_R)^n+[C_{\mathrm{dCas9},5}]^n}V_2 - \lambda[P_{\mathrm{GFP}}] \tag{79}$$

$$\frac{\mathrm{d}V_1}{\mathrm{d}t} = -\lambda V_1 \tag{80}$$

3.10 5 Identical Target Sites

$$\frac{\mathrm{d}V_2}{\mathrm{d}t} = -\lambda V_2 \tag{81}$$

$$\frac{\mathrm{d}[G_1]}{\mathrm{d}t} = \alpha_{r,G_1}V_1 - k_{C_g}[G_1][P_{\mathrm{dCas9}}] - \delta_g[G_1] \tag{82}$$

$$\frac{\mathrm{d}[C_{\mathrm{dCas9},1}]}{\mathrm{d}t} = k_{C_g}[G_1][P_{\mathrm{dCas9}}] - \lambda[C_{\mathrm{dCas9},1}] \tag{83}$$

$$\frac{\mathrm{d}[P_{\mathrm{dCas9}}]}{\mathrm{d}t} = \alpha_{p,P_{\mathrm{dCas9}}}V_2 - \lambda[P_{\mathrm{dCas9}}] - k_{C_g}[G_1][P_{\mathrm{dCas9}}] \tag{84}$$

$$\frac{\mathrm{d}[P_{\mathrm{GFP}}]}{\mathrm{d}t} = \alpha^0_{p,P_{\mathrm{GFP}}}\frac{(K_R)^n}{(K_R)^n+[C_{\mathrm{dCas9},1}]^n}\frac{(K_R)^n}{(K_R)^n+[C_{\mathrm{dCas9},1}]^n}\frac{(K_R)^n}{(K_R)^n+[C_{\mathrm{dCas9},1}]^n}\frac{(K_R)^n}{(K_R)^n+[C_{\mathrm{dCas9},1}]^n}\frac{(K_R)^n}{(K_R)^n+[C_{\mathrm{dCas9},1}]^n}V_2 - \lambda[P_{\mathrm{GFP}}] \tag{85}$$

$$\frac{\mathrm{d}V_1}{\mathrm{d}t} = -\lambda V_1 \tag{86}$$

3.11 6 Heterogeneous Target Sites

$$\frac{\mathrm{d}V_2}{\mathrm{d}t} = -\lambda V_2 \tag{87}$$

$$\frac{\mathrm{d}[G_1]}{\mathrm{d}t} = \alpha_{r,G_1}V_1 - k_{C_g}[G_1][P_{\mathrm{dCas9}}] - \delta_g[G_1] \tag{88}$$

$$\frac{\mathrm{d}[C_{\mathrm{dCas9},1}]}{\mathrm{d}t} = k_{C_g}[G_1][P_{\mathrm{dCas9}}] - \lambda[C_{\mathrm{dCas9},1}] \tag{89}$$

$$\frac{\mathrm{d}[G_2]}{\mathrm{d}t} = \alpha_{r,G_2} V_1 - k_{C_g}[G_2][P_{\text{dCas9}}] - \delta_g[G_2] \tag{90}$$

$$\frac{\mathrm{d}[C_{\text{dCas9},2}]}{\mathrm{d}t} = k_{C_g}[G_2][P_{\text{dCas9}}] - \lambda[C_{\text{dCas9},2}] \tag{91}$$

$$\frac{\mathrm{d}[G_3]}{\mathrm{d}t} = \alpha_{r,G_3} V_1 - k_{C_g}[G_3][P_{\text{dCas9}}] - \delta_g[G_3] \tag{92}$$

$$\frac{\mathrm{d}[C_{\text{dCas9},3}]}{\mathrm{d}t} = k_{C_g}[G_3][P_{\text{dCas9}}] - \lambda[C_{\text{dCas9},3}] \tag{93}$$

$$\frac{\mathrm{d}[G_4]}{\mathrm{d}t} = \alpha_{r,G_4} V_1 - k_{C_g}[G_4][P_{\text{dCas9}}] - \delta_g[G_4] \tag{94}$$

$$\frac{\mathrm{d}[C_{\text{dCas9},4}]}{\mathrm{d}t} = k_{C_g}[G_4][P_{\text{dCas9}}] - \lambda[C_{\text{dCas9},4}] \tag{95}$$

$$\frac{\mathrm{d}[G_5]}{\mathrm{d}t} = \alpha_{r,G_5} V_1 - k_{C_g}[G_5][P_{\text{dCas9}}] - \delta_g[G_5] \tag{96}$$

$$\frac{\mathrm{d}[C_{\text{dCas9},5}]}{\mathrm{d}t} = k_{C_g}[G_5][P_{\text{dCas9}}] - \lambda[C_{\text{dCas9},5}] \tag{97}$$

$$\frac{\mathrm{d}[G_6]}{\mathrm{d}t} = \alpha_{r,G_6} V_1 - k_{C_g}[G_6][P_{\text{dCas9}}] - \delta_g[G_6] \tag{98}$$

$$\frac{\mathrm{d}[C_{\text{dCas9},6}]}{\mathrm{d}t} = k_{C_g}[G_6][P_{\text{dCas9}}] - \lambda[C_{\text{dCas9},6}] \tag{99}$$

$$\frac{\mathrm{d}[P_{\text{dCas9}}]}{\mathrm{d}t} = \alpha_{p,P_{\text{dCas9}}} V_2 - \lambda[P_{\text{dCas9}}] - k_{C_g}[G_1][P_{\text{dCas9}}] - k_{C_g}[G_2][P_{\text{dCas9}}] - k_{C_g}[G_3][P_{\text{dCas9}}] - k_{C_g}[G_4][P_{\text{dCas9}}] - k_{C_g}[G_5][P_{\text{dCas9}}] - k_{C_g}[G_6][P_{\text{dCas9}}] \tag{100}$$

$$\frac{\mathrm{d}[P_{\text{GFP}}]}{\mathrm{d}t} = \alpha^0_{p,P_{\text{GFP}}} \frac{(K_R)^n}{(K_R)^n + [C_{\text{dCas9},1}]^n} \frac{(K_R)^n}{(K_R)^n + [C_{\text{dCas9},2}]^n} \frac{(K_R)^n}{(K_R)^n + [C_{\text{dCas9},3}]^n} \frac{(K_R)^n}{(K_R)^n + [C_{\text{dCas9},4}]^n} \frac{(K_R)^n}{(K_R)^n + [C_{\text{dCas9},5}]^n} \frac{(K_R)^n}{(K_R)^n + [C_{\text{dCas9},6}]^n} V_2 - \lambda[P_{\text{GFP}}] \tag{101}$$

$$\frac{\mathrm{d}V_1}{\mathrm{d}t} = -\lambda V_1 \tag{102}$$

3.12 6 Identical Target Sites

$$\frac{\mathrm{d}V_2}{\mathrm{d}t} = -\lambda V_2 \tag{103}$$

$$\frac{\mathrm{d}[G_1]}{\mathrm{d}t} = \alpha_{r,G_1} V_1 - k_{C_g}[G_1][P_{\text{dCas9}}] - \delta_g[G_1] \tag{104}$$

$$\frac{\mathrm{d}[C_{\text{dCas9},1}]}{\mathrm{d}t} = k_{C_g}[G_1][P_{\text{dCas9}}] - \lambda[C_{\text{dCas9},1}] \tag{105}$$

$$\frac{\mathrm{d}[P_{\text{dCas9}}]}{\mathrm{d}t} = \alpha_{p,P_{\text{dCas9}}} V_2 - \lambda[P_{\text{dCas9}}] - k_{C_g}[G_1][P_{\text{dCas9}}] \tag{106}$$

$$\frac{\mathrm{d}[P_{\text{GFP}}]}{\mathrm{d}t} = \alpha^0_{p,P_{\text{GFP}}} \frac{(K_R)^n}{(K_R)^n + [C_{\text{dCas9},1}]^n} \frac{(K_R)^n}{(K_R)^n + [C_{\text{dCas9},1}]^n} \frac{(K_R)^n}{(K_R)^n + [C_{\text{dCas9},1}]^n} \frac{(K_R)^n}{(K_R)^n + [C_{\text{dCas9},1}]^n} \frac{(K_R)^n}{(K_R)^n + [C_{\text{dCas9},1}]^n} \frac{(K_R)^n}{(K_R)^n + [C_{\text{dCas9},1}]^n} V_2 - \lambda[P_{\text{GFP}}] \tag{107}$$

$$\frac{\mathrm{d}V_1}{\mathrm{d}t} = -\lambda V_1 \tag{108}$$

4 Parameter Fitting

Parameters needed for simulation were either taken from previously published work on CRISPR systems [2, 3], fit to data, or hypothesized based on observed repression in the lab. The complete set of parameter base values and their origin is listed in Table S2.

Parameter	Meaning	Value	Source
$\alpha_{r,[1-6]}$	Transcription rate, for $G_1 - G_6$	$10^{3.3090}$ molecules/hour	[3]
$\alpha_{p,\text{GFP}}$	Coupled transcription and translation rate, for P_{GFP}	$10^{5.5793}$ molecules/hour	Fit to data
$\alpha_{p,\text{dCas9}}$	Coupled transcription and translation rate, for P_{dCas9}	$10^{3.0415}$ molecules/hour	[3]
δ_g	gRNA degradation rate	$10^{0.0003}$ fraction/hour	[3]
λ	Stable Molecule (Protein and Plasmid) Dilution Rate	$10^{-1.6225}$ fraction/hour	Fit to data
k_{C_g}	dCas9 and gRNA binding rate	$10^{-4.2577}$	[3]
n	Hill coefficient	0.92	[2]
K_R	Hill Repression Constant	$10^{3.95}$	Hypothesized
-	Initial Delay	18.0149 Hours	Fit to data

Table S2: Table of parameter symbols, meanings, values, and sources

The coupled transcription/translation rate of GFP, the stable molecule diffusion, and an initial lag, were all fit to an approximately 200 hour time course of Fluorolog-3 scanning fluorometer measurements that were calibrated to Molecules of Equivalent Fluoresceine (MEFL) units using flow cytometry measurements taken at the final time point. Parameters were fit using a least-squares fit for parameters in a logarithmic parameter value space in MATLAB using the ODE for the ”No gRNA control” model. The MEFL measurements for all time points and the parameterized model are shown in Figure S1.

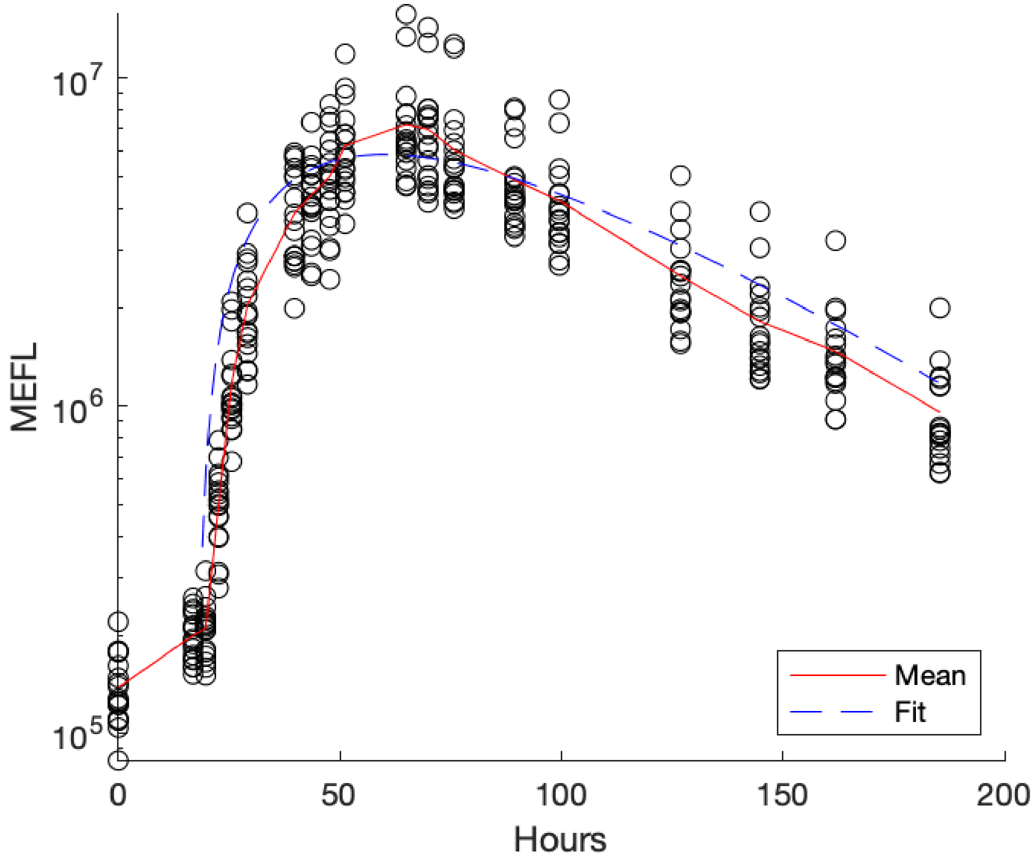

Figure S1: Experimental data and model simulation results after model fitting. A 200 hour time course of Fluorolog-3 scanning fluorometer measurements of GFP were converted to Molecules of Equivalent Fluorescine (MEFL) using a single flow cytometry measurement at the final time point. The individual data points (black circles) were used to the fit $\alpha_{p,\text{GFP}}$, λ and the Initial Delay. The results of a simulation of the "No gRNA control" model with these parameters is shown in blue. The mean value of the data points (red line) was not used directly, but is included to aid in comparison between the data and the simulation results.

The Hill repression constant (K_R) was tuned to yield a fold repression value similar to what has been seen in a published study of CRISPRi in *Arabidopsis thaliana* protoplasts [4]. In that study, a single gRNA sequence binding to a single binding site in the promoter (analogous to our one target site model) could yield fold repression ranging from 2.5 fold to 6.6 fold (Table S3). From this, we chose that a reasonable value of repression for a single target site would be 4-fold. An exploration of potential values for K_R showed that a value of $10^{3.95}$ yielded an average 4.07 fold repression over hours 72-96 in a 200 hour time course simulation (see main text, Figure 3B). A full exploration of the K_R parameter space is also shown in Figure S2.

Engineered TCTP Promoter Variant	1	4		5	
Location of gRNA Target Sites	**No binding sites**	**5'UTR**		**TATA**	
	**Rluc/Fluc**	**Rluc/Fluc**	**Fold Repression**	**Rluc/Fluc**	**Fold Repression**
**Replicate 1**	25.67491166	9.824696888	2.613303184	2.98193153	8.610161367
**Replicate 2**	27.42593024	7.97462954	3.439147875	3.681026365	7.45062043
**Replicate 3**	18.49783041	9.831309148	1.881522606	3.48866829	5.30226117
**Replicate 4**	19.85895088	8.731508876	2.274400812	3.855766344	5.150454957
**Mean**	22.8644058	9.090536113	2.552093619	3.501848132	6.628374481

Table S3: Results for Figure 1d from [4] (given in Supplementary Table 5), with additionally calculated fold repression values for each replicate.

5 Analysis of Repression Sensitivity

5.1 Sensitivity to Parameter Values

To investigate the sensitivity of each promoter architecture to each individual parameter in the model, we modulated 6 parameters while holding all other values the same. Figure S2 shows the results of these perturbations for the 1 target site model, the 4 heterogeneous target site model, and the 4 identical target site model. Each of the parameters shown in figure S2 were modulated up and down by 2 standard deviations with a 1.5-fold log-normal uncertainty, except for the stable molecule diffusion rate (λ) which was modulated down 2 standard deviations, but was only modulated up 0.75 standard deviations, since any more would cause numerical instability.

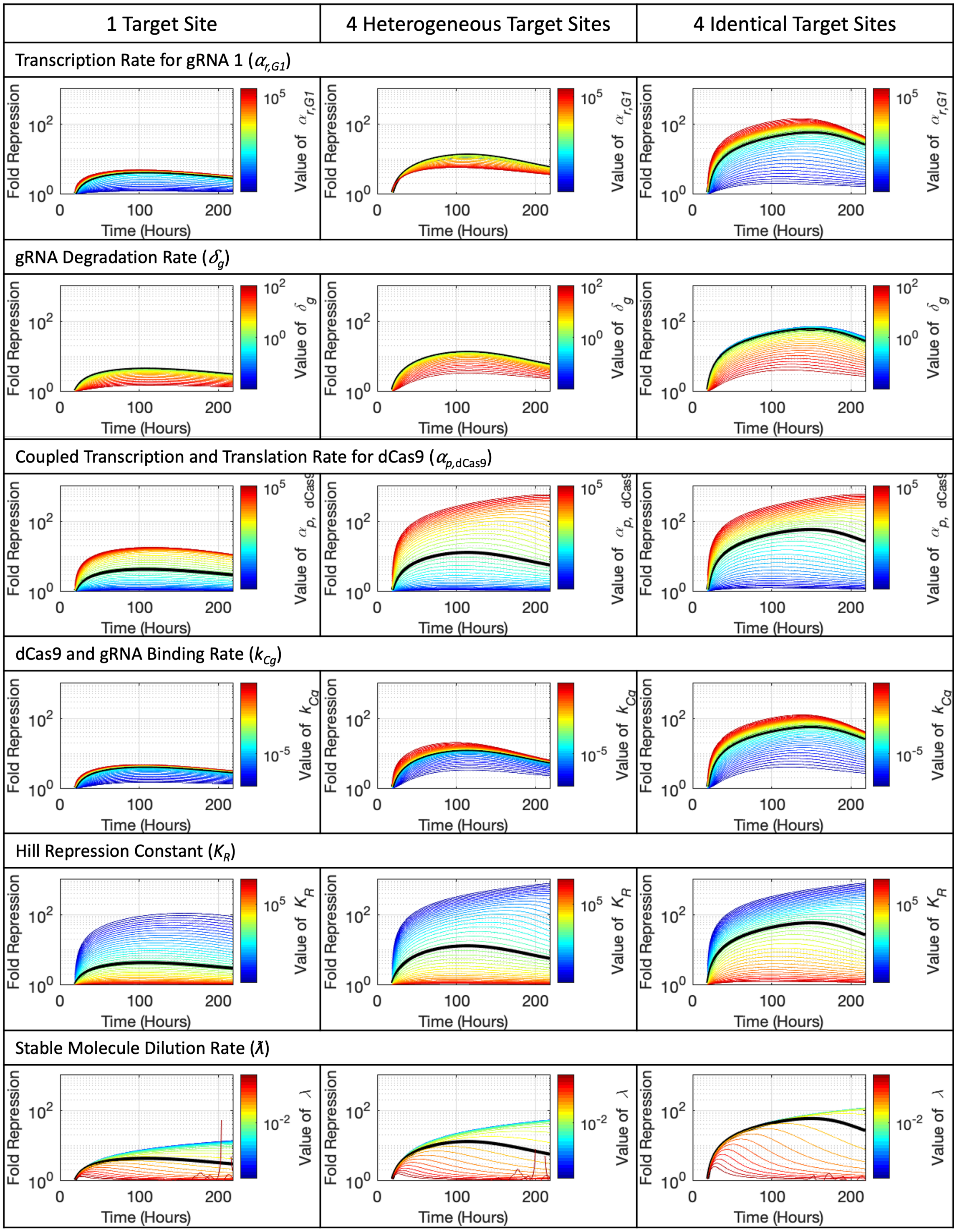

Figure S2: Examples of perturbation response for modulating individual parameters in 1 target site model, the 4 heterogeneous target site model, and the 4 identical target site model.

The modulation of most parameters show a monotonic trend, so that the lowest values (blue line) is on the opposite side of the trend of the highest value (red line). This was not the case for the α_{r,G_1} in the heterogeneous target site models, including the 4 Heterogeneous Target Site model (Figure S2 top center). In the case of this model, modulating the transcription rate of a single gRNA causes there to a different relative abundance of gRNAs in the total gRNA pool, but the total quantity of gRNA dow not become limiting because other dCas9-gRNA complexes are able to cause repression. For this reason, despite the larger range of possible fold repression values for the 4 identical target site model (Figure S2 top right) that model is not actually more sensitive to changes in the parameter value.

The remaining parameters showed similar trends for all three models, often with a similar range of fold repression values for the 4 identical target site model and 4 heterogeneous target site model, indicating that the identical target site models are not more sensitive to parameter values.

5.1.1 Multi-Parameter Perturbations

Beyond the single parameter perturbations studies, we also performed an analysis in which all parameters were simultaneously and independently perturbed. For these multi-parameter perturbations, each parameter's base value was multiplied by a log-normal random factor drawn with a standard deviation of 1.1-fold (with $\pm$ 2 standard deviations spanning a range of just under 1.5-fold). Figure S3 shows the distribution of the trajectories from 10,000 simulations of each promoter architecture, where in each simulation the model parameters have been randomly and independently perturbed. A yellow color indicates a high number of simulations have passed through that given bin, where a dark blue color indicates that no simulations have. As with the single parameter perturbations, the range of GFP values remains comparable across the different promoters, and we see no marked difference between the results from perturbation between the heterogeneous and identical target site promoters.

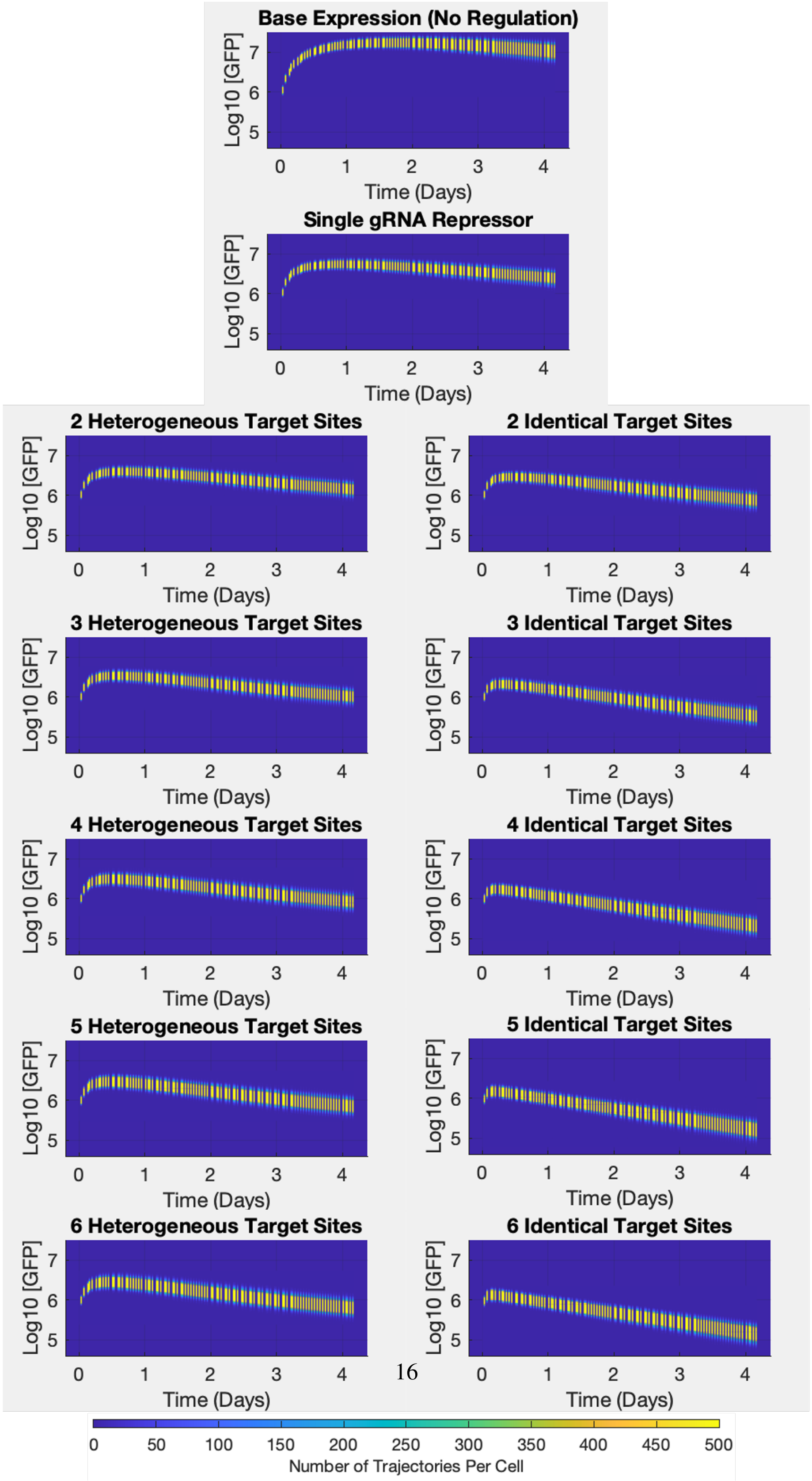

Figure S3: Distribution of trajectories generated by random perturbation of all parameters for all models.

References

[1] Uri Alon. *An introduction to systems biology: design principles of biological circuits*. Chapman and Hall/CRC, 2019.

[2] Junmin Wang, Samuel A Isaacson, and Calin Belta. “Modeling Genetic Circuit Behavior in Transiently Transfected Mammalian Cells”. en. In: *ACS Synth. Biol.* 8.4 (Apr. 2019), pp. 697–707.

[3] Helen Scott et al. “Simulation-Based Engineering of Time-Delayed Safety Switches for Safer Gene Therapies”. en. In: *ACS Synth. Biol.* 11.5 (May 2022), pp. 1782–1789.

[4] Muhammad Adil Khan et al. “CRISPRi-based circuits to control gene expression in plants”. In: *Nature Biotechnology* (2024), pp. 1–15.
